# Supplementary figures and images for: Spatial and temporal activity patterns of Golden takin (Budorcas taxicolor bedfordi) recorded by camera trapping
Source: PeerJ. 2020 Nov 26;8:e10353. doi: 10.7717/peerj.10353 (PMC7700736; doi:10.7717/peerj.10353)

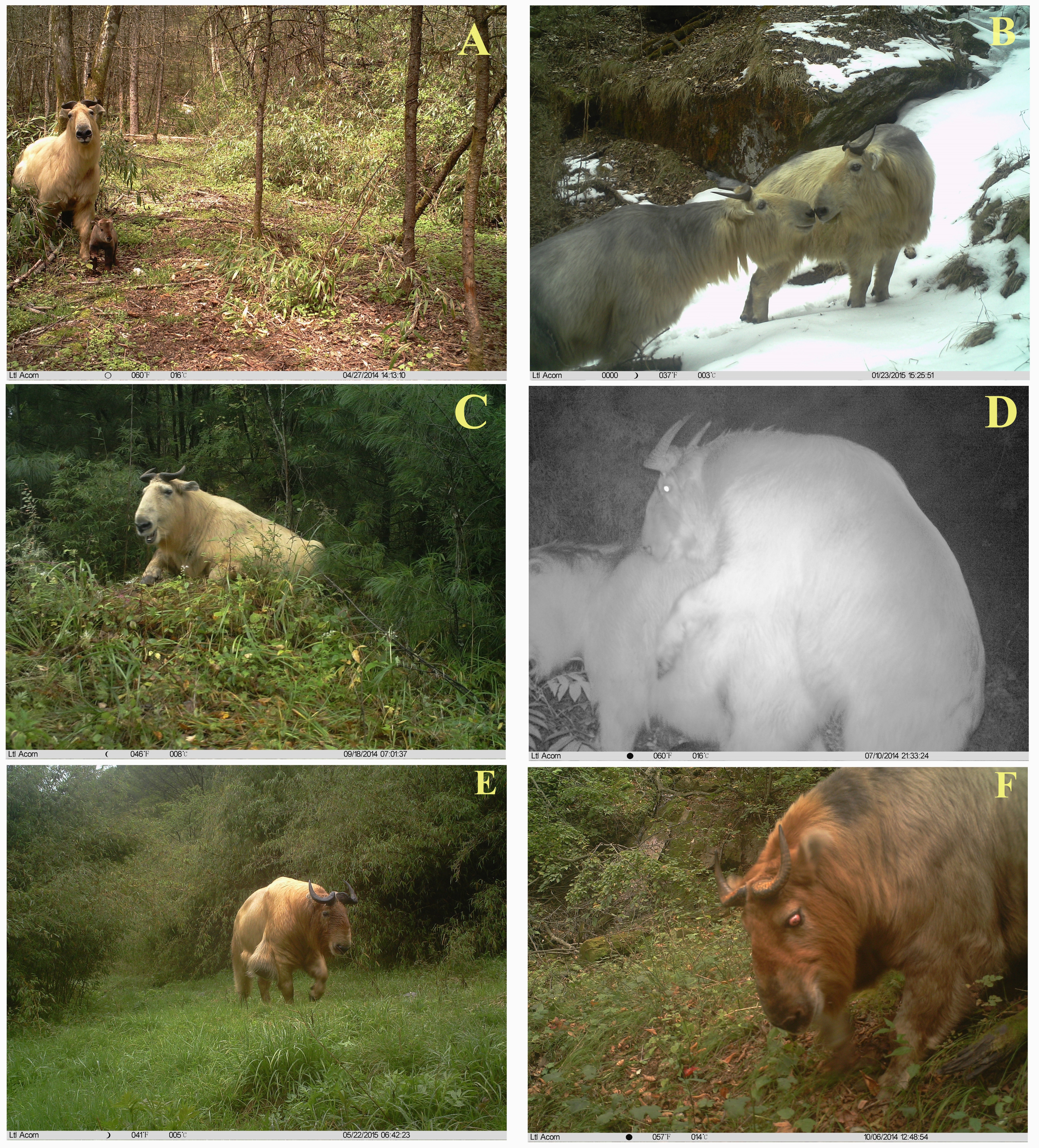

Supplement: Supplemental Information 2 — (A) golden takin’s calf with adult takin; (B) social interaction of golden takins; (C) golden takin rests while ruminating; (D) male and female golden takins engage in mating behavior; (E) golden takin with tumor; (F) golden takin with eye abnormality [file peerj-08-10353-s002.jpg]
